# “Girls on the Move” intervention protocol for increasing physical activity among low-active underserved urban girls: a group randomized trial

**DOI:** 10.1186/1471-2458-13-474

**Published:** 2013-05-15

**Authors:** Lorraine B Robbins, Karin A Pfeiffer, Amber Vermeesch, Kenneth Resnicow, Zhiying You, Lawrence An, Stacey M Wesolek

**Affiliations:** 1 College of Nursing, Michigan State University, 1355 Bogue Street, East Lansing, MI 48824, USA; 2 Department of Kinesiology, College of Education, Michigan State University, 27R IM Sports Circle, East Lansing, MI 48824, USA; 3 School of Public Health, University of Michigan, 1415 Washington Heights, Ann Arbor, MI 48109, USA; 4 Center for Health Communications Research, University of Michigan, 2800 Plymouth Road, Ann Arbor, MI 48109, USA

**Keywords:** Group randomized trial, Physical activity, Adolescents, Females, School, Intervention, Tailored counseling, Motivational interviewing

## Abstract

**Background:**

Increasing moderate to vigorous physical activity among urban girls of low socioeconomic status is both a challenge and a public health priority. Physical activity interventions targeting exclusively girls remain limited, and maintenance of moderate to vigorous physical activity during the post-intervention period has been difficult to maintain. The main aim of the 5-year “Girls on the Move” group randomized trial is to evaluate the efficacy of a comprehensive school-based intervention in increasing girls’ minutes of moderate to vigorous physical activity and improving cardiovascular fitness, body mass index, and percent body fat immediately post-intervention (after 17 weeks) and at 9-month post-intervention follow-up (9 months after end of intervention).

**Methods/Design:**

A total of 24 urban middle schools in the Midwestern U.S. will be randomized to either receive the intervention or serve as a control (N = 1200 girls). The intervention, based on the Health Promotion Model and Self-Determination Theory, will include: (1) two face-to-face motivational, individually tailored counseling sessions with a registered nurse, one at the beginning and the other at the end of the intervention period; (2) an interactive Internet-based session during which each girl receives individually tailored motivational and feedback messages via iPad at 11 weeks (shortly after midpoint of intervention); and (3) a 90-minute after-school physical activity club. Racially diverse, low-active, 10- to 14-year-old 5^th^ to 8^th^-grade girls will complete questionnaires and physical measures at baseline and post-intervention (n = 50 per school). Minutes of moderate to vigorous physical activity will be assessed with accelerometers. Cardiovascular fitness will be assessed by estimating VO_2_ max with PACER (Progressive Aerobic Cardiovascular Endurance Run) scores. Height and weight will be assessed to calculate body mass index. Percent body fat will be estimated with a foot-to-foot bioelectric impedance scale. Linear mixed effects regression analyses will be performed to assess intervention effects.

**Discussion:**

This multi-component approach is expected to improve girls’ moderate to vigorous physical activity and related physical outcomes.

**Trial Registration:**

ClinicalTrials.gov Identifier NCT01503333

## Background

By 9^th^-grade, only about 22% of girls meet national recommendations of at least 60 minutes of physical activity (PA) every day, and almost 28% are already overweight or obese [1]. An estimated 49% of overweight adolescents and 61% of obese adolescents, ages 12 – 19, have at least one cardiovascular disease (CV) risk factor in addition to their weight status. Among those having a normal weight, approximately 37% have at least one CV disease risk factor [2]. These findings indicate that all girls, regardless of weight status, would benefit from involvement in health-promoting programs, such as those designed to increase PA.

A decline in PA with advancing age across adolescence [3] is related to increases in girls’ body mass index (BMI) and adiposity [4-7]. Girls who are overweight or obese in adolescence are at an increased risk for co-morbidities in adulthood [8,9] and premature mortality [10]. An urgent need exists to intervene before high school is reached to help girls establish a pattern of attaining adequate daily PA as a means to reduce their risk for these adverse health outcomes. Our intervention is directed toward increasing moderate to vigorous physical activity (MVPA) among girls in 5^th^ through 8^th^ grade. Because declining PA is particularly evident among urban, minority girls of low socioeconomic status (SES) [11,12] and increasing their MVPA is a vital public health priority [13], we chose to focus our intervention on this group.

Our intervention extends beyond prior approaches by including two evidence-based strategies offered during or after the school day: Internet-delivered individually tailored counseling and face-to-face motivational interviewing from a registered nurse. Prior studies have used either computerized individually tailored advice [14-17] or motivational interviewing alone [18,19] to increase MVPA among adolescents with limited success. However, Internet-delivered approaches appeal to this age and those that are motivational, theory-based, and individually tailored hold promise as a means for increasing MVPA [20]. Some face-to-face communication is recommended because of its intimacy and rapport [21] and the inherent ability to address individual motivational and emotional factors and barriers not detectable via questionnaires. To our knowledge, no other intervention has directly involved nurses in providing motivational interviewing to promote MVPA among girls, even though nurse-delivered motivational interviewing during school hours has been found to be effective for improving other adolescent behaviors (e.g., reducing smoking) [22,23]. In addition to these two strategies, the intervention provides opportunities for PA (e.g., PA club) after school three days per week with bus transportation home. Because of the integration of these recommended, albeit diverse, strategies and the potential for a synergistic effect, our multi-component intervention is both novel and potentially potent.

### Aims and hypotheses

The main study aim is to evaluate the efficacy of our comprehensive 17-week school-based intervention in increasing girls’ minutes of MVPA and improving CV fitness, BMI, and percent body fat immediately post-intervention (after 17 weeks) and at 9-month post-intervention follow-up (F/U; 9 months after end of intervention). The primary outcome is minutes of MVPA measured after 17 weeks. The intervention includes three components, two of which are individual-level: 1) two face-to-face motivational, individually tailored counseling sessions with a registered nurse, and 2) an interactive Internet-based session during which each girl receives individually tailored motivational and feedback messages (11 weeks). One group-level component, a PA club led by PA instructors (e.g., individuals from community who have experience conducting youth physical activity programs; teachers, including physical education; and sports team coaches), provides an important venue after school to assist girls in establishing a behavioral pattern of MVPA. Girls receiving the intervention will be compared to those in control schools having usual school offerings, some of which may include physical education.

Specific hypotheses include:

•Immediately post-intervention, minutes of MVPA (measured by accelerometer) will be greater by 16 minutes/week among girls in intervention, as compared to control, schools.

•At 9-month post-intervention F/U, minutes of MVPA will be greater among girls in the intervention than control schools.

•Immediately post-intervention and at 9-month post-intervention F/U, CV fitness will be higher and BMI and percent body fat will be significantly lower among girls in the intervention than control schools.

A secondary objective is to examine if MVPA changes are mediated by cognitive (e.g., perceived benefits of PA, barriers to PA, PA self-efficacy, social support) and affective variables (e.g., enjoyment of and motivation for PA).

## Methods/Design

### Study design

The Girls on the Move intervention is a group randomized trial involving 24 racially diverse, urban schools in low SES areas in the Midwestern U.S. In years 2, 3, and 4 of the 5-year study (2011–2016), 8 schools will be randomly assigned to either receive the intervention (n = 4/year) or serve as the control condition (n = 4/year). The research will be reported in accordance with Consolidated Standards of Reporting Trials (CONSORT) recommendations [24].

In order for their school to be included in the study, school administrators must be willing to accept the randomization status of the school and sign a memorandum of understanding. Schools will be included in the study based on the following criteria: (1) location in urban community setting; (2) enrollment greater than 100 girls in each school or more than double the number of girls needed for the study site (n = 50 per school) in any combination of 5^th^, 6^th^, 7^th^, and/or 8th-grade; and (3) student body comprised of at least 50% minority versus non-minority race or ethnicity and including a similar percentage enrolled in the free and reduced lunch program. Schools were excluded if administrators were not interested in participating, did not agree to random assignment, or could not guarantee their availability at 9-month post-intervention F/U.

To avoid any bias, schools are paired prior to randomization based on the following criteria: school type (e.g., academic grades offered), school size, percentage of students who are white vs. non-white race, and proportion of students receiving the free and reduced lunch. Prior to the start of the school year, the PI and project manager inform each principal regarding the randomization status of his or her respective school to assist each principal with future planning. Principals are told that the randomization information must remain confidential until completion of baseline data collection in the fall.

The project’s measurement coordinator trains staff according to standardized protocols and certifies each one for data collection only after successful practice demonstrations. The measurement coordinator or project manager oversees all sessions to ensure fidelity to the measurement protocol. The same group of trained staff collects data for each cohort or matched pair including an intervention and control school over the same three- or four-week period. Only half of the data is collected from half of the girls at each paired school during the first week. The remainder of the data is collected from the same wave of girls during either the second or third week. Half of the data is obtained from the other half or wave of girls at each school during the second or third week with the remaining half of the data being collected during the following week (week three or four, respectively). Whether the data collection spans across three or four weeks for each pair of schools is determined based on requests from school administrators. To reduce the potential of non-typical PA occurring during a single week for all girls at a particular school site, two waves are used during all data collection periods in every participating school. Obtaining half of the data, as opposed to all, at a single time point also reduces the response burden for the girls and decreases the time needed during a school day. Complete data are collected at pre-intervention/baseline and immediately post-intervention (after 17 weeks). At 9-month post-intervention F/U, only PA is measured (via accelerometer) to assess for any long-term effects. For completing the survey data at each time period, girls receive an incentive, such as a T-shirt or a drawstring backpack, with the Girls on the Move logo. For wearing the accelerometer as instructed for one week at each time period, girls receive a $20.00 gift card to a local general store. If the data are inadequate due to limited wear-time and the accelerometer needs to be re-worn, girls receive a reduced gift card amount of $10.00 after the second week of wearing it.

After all baseline measures are obtained in a school, members of the research team contact the parents and girls by phone to inform them of their school’s randomization status. The measurement and intervention teams function independently so as to blind members of the former group to each school’s randomization status throughout the entire study. Girls, parents/guardians, principals, nurses, and school staff are not told about hypotheses.

### Participants, eligibility, and recruitment

The researchers received ethical approval to conduct the study from the University Biomedical Institutional Review Board. School administrators also provided approval to conduct the study in their respective districts. At the beginning of each school year, the project manager schedules mutually convenient times for one or two members of the research team to meet with girls called to an assembly for the sole purpose of discussing the study in each of the eight schools. During the meeting, the researchers share information about the study and invite girls to participate. Girls are informed that their school will be randomly assigned to either receive an after-school PA club called Girls Only Activity for Life (G.O.A.L.) or continue with usual school offerings. They are told that girls in all schools will have the opportunity to receive incentives for participating in data collection activities, called “download days,” in the fall and spring and then again in the following school year. In addition to the brief verbal overview of the study, the researchers play an attractive two-minute recruitment video created by the research team in collaboration with a local production company. The video highlights reasons to participate (e.g., no financial cost and opportunity to make or be with friends) and includes short scenes of girls having fun during various study phases, such as data collection. For example, the video shows girls wearing attractive, colorful headphones as they respond to survey questions using an iPad with voiceover.

Following the video presentation, the researchers answer questions and distribute packets containing study materials to interested girls. Each packet includes a consent/assent form and screening tool (e.g., number of days/week girl is obtaining 60 minutes of MVPA; number of days interested in attending PA club; school or community sports or other organized PAs girl is participating in). Girls are told if they return the completed forms to the researchers present at their school during the next day or two, they will immediately receive a $5.00 cash incentive, regardless of whether they are interested in participating or not. Response rates are recorded. During the first year of recruitment (fall 2012), this strategy exceeded expectations with 1,115 girls returning completed packets, 832 of whom were interested in participating and provided written assent and parent/guardian permission. However, 178 of the 832 (21.4%) girls did not qualify based on the inclusion criteria.

Girls meeting the following inclusion criteria are selected for participation on first come, first served basis: (1) 5^th^- thru 7^th^-grade girls (ages 10–14; 8^th^-graders if needed in schools having only 7^th^- and 8^th^- grades); (2) available and willing to participate in the PA club 3 days/week for 17 weeks; (3) available for F/U (9 mos. after intervention ends); (4) agree to school random assignment; and (5) able to read, understand, and speak English. Exclusion criteria are: (1) Involved in or planning to be involved in school or community sports or other organized PAs, such as dance lessons, that involve MVPA and require participation 3 or more days/week after school; and (2) a health condition precluding safe MVPA. Girls getting 3 or more days/week of organized MVPA after school are excluded because they are already involved for the same number of days that would be available to them to engage in MVPA in the PA club.

If a girl begins the study and later decides to participate in a sport (school requirements usually equal an average of 4 days/week) or other organized PA after school, she can continue in the study and complete the nurse and Internet-based computer sessions. The nurse will discuss the MVPA recommendations. The girl will receive encouragement to be physically active from the nurse and via the computer. If the girl stops participation in the sport or organized PA, she will be encouraged to return to the PA club 3 days/week. Study personnel will carefully monitor and track all participation.

### Girls on the move intervention

#### Pilot study

The Girls on the Move intervention was developed and refined based on a pilot project, which demonstrated the feasibility of implementing an after-school PA program for girls at their school and the value of involving girls in face-to-face motivational interviewing sessions delivered by a registered nurse [25].

#### Theoretical underpinnings

The Girls on the Move intervention involves a novel approach based on the integration of the Health Promotion Model (HPM) [26] and Self-Determination Theory (SDT) [27]. The HPM purports that personal factors and various prior behaviors are associated with MVPA, but personal factors (e.g., age, academic grade, race or ethnicity, SES, and developmental stage), with the exception of CV fitness, are not amenable to intervention. Yet, personal factors and various prior behaviors can influence cognitive and affective mediating variables, including perceptions of benefits (HPM) [28], barriers (HPM) [28-30], self-efficacy (HPM) [28-32], social support (HPM) [28,29,31,32], enjoyment (HPM) [33], and motivation (SDT), which also affect MVPA, but are modifiable [28]. Each component incorporates three basic needs proposed by SDT for promoting autonomous motivation to drive behavior: competence, autonomy, and relatedness. Each of the three intervention components are designed to address the cognitive and affective mediating variables and the basic needs.

#### Training for intervention delivery

Prior to starting the intervention, all interventionists are required to complete the online university protection of human subjects program. The PA club instructors attend a 4-hour session prior to the start of the intervention followed by a 6-hour booster session that occurs shortly before the midpoint of the intervention. The intervention coordinator conducts both sessions and also meets with the PA club instructors at each school for at least 1 hour every month throughout the entire intervention or more frequently if needed to discuss any issues and reinforce policies and procedures.

The nurses attend two 8-hour days of training in motivational interviewing for increasing PA, conducted by a member of the Motivational Interviewing Network of Trainers. Sessions are conducted on four different weekend days to accommodate the nurses’ schedules. Training includes content on the study’s theoretical underpinnings, adolescent development, and adolescent-centered counseling (e.g., reflective listening), as well as motivational interviewing demonstrations. Nurses are given ample time to practice techniques and receive feedback. The trainer guides nurses to elicit change talk (expressions indicating a need to increase MVPA), resolve ambivalence, and encourage individuals to generate their own solutions. Role-playing occurs until the trainer notes proficiency. All nurses receive policy and procedure manuals detailing specific responsibilities.

#### Components

##### Two face-to-face motivational, individually tailored counseling sessions

During the beginning of the intervention period or within four weeks after completing the baseline measures, each girl engages in a 20-minute motivational, individually tailored counseling session with the nurse at the time of the PA club or during the school day. In the pilot study, a 20-minute time period was reasonable during the school day and adequate for achieving session objectives. A face-to-face approach was selected to provide an opportunity for the nurse to personally connect with (relatedness) and individually assist each girl in overcoming any unique or newly arising negative personal issues interfering with her PA or club attendance (e.g., another girl in PA club made rude comment to girl).

In this initial session, the nurse uses a one-page printout of each girl’s key responses gleaned from the baseline survey, as a guide to tailor the counseling or stimulate discussion (e.g., girl not actively engaging), while applying the motivational interviewing communication style. For example, the nurse will tailor the counseling by identifying the girl’s top benefits (listed on printout) for her and asking her to “tell me more” about the benefits as a means to help the girl reinforce their importance (motivational interviewing strategy). A similar approach and communication style is used for other theory- or model-related variables. To promote competence, relatedness, and autonomy (respectively), the nurse will: help each girl identify her strengths (abilities) to increase her MVPA; demonstrate empathy; allow for some choice regarding what is discussed (e.g., shared agenda setting). The nurse will assist each girl to work through ambivalence and resistance surrounding change by helping her think about and verbally express her own reasons for and against change and how her behavior can affect the ability to achieve life goals or fulfill core values [34,35]. An important objective for the nurse during this initial session involves exploring ways for the girls to attend the club regularly and supplement MVPA on their own outside the club and overcoming resistance.

During the final month of the intervention, girls will engage in another 20-minute session with the nurse. In this second session, the nurse uses a one-page printout of each girl’s key responses gleaned from the completed baseline and midpoint survey, as a guide to tailor the motivational interviewing in a manner similar to the first session. The nurse will also encourage each girl to discuss ways to attain adequate PA after the club ends.

##### Interactive Internet-based session via iPad

At 11 weeks or shortly after the midpoint of the intervention, girls use the iPad to complete an abbreviated survey comprised of items similar to those the girls answered at baseline. Each girl receives individually tailored motivational and feedback messages based on her survey responses, including acknowledging changes since baseline. Messages are delivered via voiceover from one of four young, racially diverse female models that the girl selected via the iPad at baseline. A graphic artist created the four models and modified their physical features as needed based on evaluative feedback from the research team and 6 racially diverse girls who were similar in age to the participants. Following selection, each girl has periodic exposure to the chosen model via the iPad (relatedness). Models emphasize that girls of “their” age can choose PAs they want to do from many existing options (autonomy) and that each girl is capable of developing PA-related skills (competence) and attaining national PA recommendations.

#### After-school PA club

To assist girls in recognizing benefits and overcoming barriers to PA, girls participate in a 90-minute after-school PA club 3 days/week. The club offers enjoyable PAs designed for girls to help them improve PA skills [36] (competence; self-efficacy) with social support from the peer group and instructors (relatedness). The variety of PAs offered helps them choose what to do outside the PA club. Girls will also have opportunity to provide some input regarding PA club activities (autonomy).

The 90-minute PA club includes: 10 minutes for organizational tasks and healthy snack; 5 minutes for warm-up including stretching; a 60-minute opportunity for MVPA; 5 minutes for cool-down including stretching; and 10 minutes for organizational tasks and healthy snack. An important, added benefit is that the club may prevent girls from engaging in sedentary activity and high-calorie snacking at home after school, while parents/guardians may still be at their place of employment. The PA club instructors encourage the girls to supplement MVPA on their own outside the club to achieve the national PA recommendations.

### Measures

#### Outcomes

Minutes of MVPA are assessed via ActiGraph GT3X-plus, a small, lightweight accelerometer (http://www.theActiGraph.com) [37] that is reliable and valid for assessing MVPA [38,39]. It records acceleration counts from which minutes of MVPA will be estimated [39]. A trained staff member provides and reviews instructions on wearing the accelerometer with each girl and plays a two-minute video, created by the research team in collaboration with a local production company, for the purpose of reiterating the importance of wearing the monitor. Girls are told to wear the monitor attached to an elastic belt on their right hip from the time getting out of bed in AM to the time returning to bed to sleep at night for seven consecutive days, but not when bathing or swimming. To remind them to wear it, girls receive an automated phone call to their homes every morning before school and at 11 A.M. on Saturday and Sunday until the monitor is returned to school. Monitors are initialized and set to begin data collection at 5:00 A.M. on the day after they are distributed to girls at school. Data are collected and stored in raw format, as this new ActiGraph model has high storage capacity and allows choice of time sampling interval (or epoch length) after data are collected and downloaded. This way, the research team may apply pattern recognition techniques to data if desired.

Although different epoch levels can be selected, initial analysis will be conducted using count cut-points (15-second epochs) created by Evenson and colleagues [40]. Prior research by one of the study authors showed these cut-points have the best sensitivity and specificity when compared to others [41]. Count thresholds will be used for activity intensities: moderate (574–1002 counts/15 seconds) and vigorous PA (≥1003 counts/15 seconds). Fifteen-second increments with counts at or above 574 will be summed from 6 A.M. to midnight to determine minutes of MVPA. Data will be excluded if the monitor is taken off during waking hours (noted by 20 or more consecutive minutes of continuous zeros - not observed in an awake child wearing an accelerometer) [42]. Guided by the 80% rule, we plan to use data on participants who provide at least 4 days of data (a significant increase in reliability occurs at 4 days). On weekdays and weekends, we expect monitors to be worn 11.2 hours and 7.2 hours, respectively (adolescents are expected to be awake 14 hours/day on weekdays and 9 hours/day on weekends so 80% corresponds to 11.2 and 7.2 on weekdays and weekends, respectively) [43].

Cardiovascular fitness is determined using the Progressive Aerobic CV Endurance Run (PACER) [44], a 15-meter or 20-meter shuttle run, that estimates aerobic capacity and CV endurance. No more than six participants at a time run from one line to another on a flat surface, according to audio cues. Each time participants complete a run in one direction, it is considered completion of one lap. The distance (15 versus 20 meters) of one lap is determined by space available. The pace of the audio cues increases with time until participants can no longer complete the laps in the time allotted. Staff members record number of laps completed for use in analysis (higher number of laps = greater CV fitness) [44]. Students are considered finished with the test when they have not completed two laps within the allotted time. Number of laps completed is converted to estimated VO_2_ for analysis. PACER has been used with overweight adolescent girls [45]. It avoids singling out lesser fit individuals by allowing them to finish first rather than last with everyone watching.

Body mass index and percent body fat are measured behind a privacy screen. Height without shoes is measured to the nearest 0.1 cm with a Shorr Board (Shorr Productions, Olney, MD). Weight is assessed to the nearest 0.1 kg and percent body fat is measured to the nearest 0.1% using a foot-to-foot bioelectric impedance scale (Tanita Corporation, Tokyo, Japan). BMI raw scores (weight in kg/height in meters squared) are calculated; z-scores and percentiles for age are determined using a SAS program for CDC Growth Charts, available online from the National Center for Chronic Disease Prevention and Health Promotion.

#### Cognitive and affective variables

Perceived benefits of and barriers to PA are measured with two scales: a 12-item Perceived Benefits Scale and a 17-item Perceived Barriers Scale. Cronbach’s alphas for a 10-item Perceived Benefits Scale and a 9-item Perceived Barriers Scale developed by the first author in prior research with adolescents were .80 and .78, respectively [46]. Face, content, and construct validity has also been reported for each scale [46]. Both have response choices ranging from *not at all true* (coded 0) to *very true* (coded 3). Prior to the pilot and this current study, the investigators added new items to the existing instruments based on recommendations that were received from 25 6^th^- through 8^th^- grade girls participating in focus groups conducted by the first author. In the pilot study, the additional items resulted in Cronbach’s alphas of .85 and .88 at baseline and .84 and .86 at post-intervention for the Perceived Benefits and Barriers Scales, respectively [25]. Individually tailored motivational and feedback messages delivered via the iPad are based on each girl’s responses to scale items related to both mediating variables. Each girl’s top benefits and barriers are also reported on the one-page printout provided to the nurse for use during the motivational interviewing sessions.

Enjoyment, another mediating variable, is assessed with the PA Enjoyment Scale [47], which has demonstrated factorial and construct validity when used with adolescents [48,49]. To decrease the overall response burden and length of the original 16-item instrument, create an equal balance between negatively and positively worded items, and exclude double negatives found to be misunderstood by girls in the prior pilot work [25], three positively worded and three negatively worded items were selected for inclusion in this study. Negatively worded items (e.g., I feel bored) are reverse scored. Four response choices range from *not at all true* to *very true*. In the pilot study, Cronbach’s alphas were .78 and .81 at baseline and post-intervention, respectively, for the 6-item scale [25]. Each girl’s responses to the scale items were provided for the nurse.

Social support received for PA is measured via an 8-item Social Support Scale. One item example is: *Someone encourages me to exercise.* A 5-item scale was used in the past by the first author [50]. The items in the 5-item scale were based on two scales developed by other researchers to assess parent and peer support [51,52]. Prior to the pilot study, the first author modified the scale to increase item clarity and focus on support received from people in general or as a whole in the participant’s life. To increase the comprehensiveness of the scale, three items were added. The additional three items and refinements were also based on evaluative feedback from girls in the focus groups conducted by the first author. Four response choices included: *Never* (coded 0), *Rarely* (coded 1)*, Sometimes* (coded 2), and *Often* (coded 3). Higher scores indicate greater social support and vice versa. In prior work, scale items assessing encouragement and provision of transportation from others, were found to be related to PA [50]. In the pilot study, the 8-item scale had a Cronbach’s alpha of .93 [25]. Responses related to this mediating variable, such as sources and forms of social support, were used to tailor the iPad-delivered messages and also shared with the nurse via the one-page printout.

PA self-efficacy is measured with two instruments in order to capture different dimensions of the mediating construct. One instrument is used to determine how much girls agree that they can be active in their free time when facing barriers or not. The instrument was originally developed by Saunders and colleagues [53] and was later modified to include 8-items with five response choices ranging from “disagree a lot” to “agree a lot” [48]. Test-retest reliability in 6^th^ and 8^th^ grade girls was .84 [48,54]. In this study, because social support was already being assessed, two items focusing on the girl’s ability to ask others to be active with them were deleted. Also, the middle response choice, “neither agree nor disagree” included in the 8-item measure was deleted in the remaining 6 items.

The second measure of self-efficacy assesses how sure girls are of their ability to adhere to exercising whether or not barriers exist. The items of the resisting relapse factor of the Self-Efficacy for Exercise Behaviors Scale [55] developed for adults were modified by other researchers for the Active by Choice Today (ACT) trial [56]. Because two of the original 12 items were deemed irrelevant, they were deleted, and only 10 items were used in the ACT trial [56]. Reliability and construct validity are reported elsewhere [57,58].

Motivation is measured with the Behavioral Regulation in Exercise Questionnaire – 2 (BREQ-2). The 19-item scale for measuring this mediator was modified to delete six redundant items and three items that were similar to those included in the enjoyment scale. Reducing the number of redundant items was essential to reduce the overall survey length and response burden for this young age group. Some minor wording changes were made to aid in comprehension. Response choices range from “not true” to “very true.” In one study with adolescents, Cronbach alpha coefficients for the five subscales associated with the BREQ-2, including amotivation, external regulation, introjected regulation, identified regulation, and intrinsic motivation, were 0.82, 0.76, 0.74, 0.74 and 0.87, respectively [59]. A more recent study showed adequate factorial validity and moderate to high internal consistency reliability for the five subscales with Cronbach alpha values ranging between 0.61 and 0.88 [60].

#### Personal factors and behaviors

Single items are used to assess the following personal factors and behaviors: number of days attending PE or gym class in a typical week in the current semester or quarter; participation on a sports team or in classes/lessons (dance, martial arts, gymnastics, tennis) at school or outside school; number of hours watching TV or movies, playing video games or using the Internet for something that is not schoolwork, and talking on the phone or sending text messages. SES is determined by an item listed on the consent form that asks parents whether or not their child participates in the free and reduced price lunch program. Age, academic grade, race and ethnicity are obtained from items listed on the consent form or screening tool.

Each girl’s stage of development is assessed with the Pubertal Development Scale [61]. Alpha coefficients are .68 - .83 [61]. Pender et al. found agreement between observations of participants' developmental characteristics and self-report to be >90%. In other studies, correlation coefficients comparing observed developmental characteristics and self-reported scores were as high as .79 [61]. Girls rate themselves on growth spurt, body hair (underarm), skin changes, and overall development as compared to other girls of similar age. Response choices will be: (1) no; (2) yes, barely; (3) yes, definitely; or (4) development complete. Girls respond to questions about breast growth and menstruation. Scores for body hair, breast growth, and menarche (1 = no menstruation yet; 2 = yes, menstruation started) are combined to determine developmental stage (early, middle, or late puberty).

### Process evaluation

#### Internet-based program evaluation

During the first year of the five-year study and prior to recruitment, the University of Michigan Center for Health Communications Research designed the Internet-based program and created an administrative console with databases for tracking and monitoring all day-to-day study operations. The console houses all evaluative information.

Also, in the first year of the study, nine racially diverse 4^th^ - 8^th^ grade girls from an urban school and 10 4^th^ grade girls from a Boys and Girls Club in the same community evaluated the Internet-based program, which included the survey and feedback messages, for usability, acceptability, and comprehension. They were asked to: rate the program using an instrument designed by the research team (e.g., complexity; degree that program adheres to HPM and SDT constructs); comment about its interactivity, speed, and cultural and developmental appropriateness (e.g., whether or not it is fun, exciting, interesting, acceptable, and appealing to all racial groups and girls of middle school age); and provide suggestions for improvement. The program was refined based on their feedback.

#### PA club evaluation

PA club instructors use the Internet-based program to record reach (e.g., girl’s attendance and punctuality at PA club). To assist the researchers in evaluating dose, one of the PA club staff members serving as the club manager records the types of activities offered for the day, including whether healthy snacks were distributed.

The second author trained four process evaluators to evaluate the PA club via an observation tool and a quantitative checklist with rating scale in the first mo. (week 3), at the midpoint (week 9), and near the end (week 15). The checklist includes items for evaluating dose delivered or staff adherence to specific areas associated with the PA club protocol and fidelity or the degree that the PA club reflects important theoretical concepts of the HPM (e.g., enjoyment - “fun” PAs offered) and SDT (e.g., autonomy, relatedness, competence). A process evaluator evaluates the entire session for one day during one week, and another process evaluator evaluates the session on another day during the same week. They report to the second author at the end of each week and discuss their findings. The second author or intervention coordinator intervenes as needed with the PA club instructors to promote interventionist consistency in adhering to the protocol.

Five girls per school are randomly selected every other week to wear the ActiGraph GT3X-plus to measure the girls’ level of PA during the club time. To determine dose received, data are downloaded at the end of each week to a computer. A research team member (e.g., intervention coordinator, project manager, principal investigator, co-investigator) randomly monitors a session at each club at least weekly to evaluate adherence to protocols and retrain staff as needed. During the middle of the first half of the intervention, the manager contacts parents/guardians of all girls who missed a week (all 3 days) or more than an average of two PA club sessions in a week over the initial three 3-week period to explore ways to help the girls attend the club and attain adequate PA.

#### Nurse counseling evaluation

To assist the research team in evaluating reach and dose, the nurse uses the Internet-based program to record each girl’s attendance at and date of the counseling session, plus the start and end time of the session. For girls who miss a week (all 3 days) or more than an average of two PA club sessions in a week, the nurse records reasons for not attending the PA club after each girl’s counseling session. All sessions for assented participants who have written parental/guardian permission are audio-taped with a portable digital recorder. In the pilot, we found that giving the nurse a recorder to tape sessions was more efficient than randomly selecting a few sessions for audio-taping and having research assistants traveling to the school to record them. Also, this approach ensures that all sessions are being delivered as planned. The nurse will allow each participant to consent or refuse to be audio-taped at the beginning of each session. After the nurse conducts approximately 6 sessions, the first author meets with the nurse to evaluate one of the audio-taped sessions together (in collaboration) with the nurse and reinforce motivational interviewing principles.

To further determine adherence to the counseling protocol and motivational interviewing principles, our statistician randomly selects two audiotapes from those recorded by each nurse in the first session to be transcribed verbatim. Following evaluation of the transcripts and prior to the second session, the first author meets and discusses the two selected cases with the respective nurse on an individual basis to assist the nurse in adhering to the technique to avoid drift and ensure interventionists’ consistency, troubleshoot, and offer strategies for enhancing motivational interviewing skill transfer during the final session. During the middle of the second half of the intervention, the nurse contacts all parents/guardians to encourage them and explore ways to help their daughter continue her physical activity after the club ends.

The first and third author will evaluate the two audio-recordings and transcripts from the first session and two from the second session at each of the four intervention schools per year. Similar to the pilot study, the Motivational Interviewing Treatment Integrity (MITI) Code will be employed [63]. Both authors have expertise in using the MITI, a reliable, valid, and sensitive scale for evaluating motivational interviewing fidelity [63,64].

### Evaluation of intervention components - immediately post-intervention

Girls in the intervention group will evaluate each intervention component (nurse counseling, Internet-based program, and the PA club) by responding to the Girls on the Move Intervention Evaluation Instrument (developed for the pilot study) that will be integrated into the Internet-based program. The nurse and PA club instructors will complete a self-evaluation form that will be compared with the girl’s evaluations. Descriptive statistics will be obtained. Matched pairs t-tests will be used to compare the data. A research team member will share evaluative findings with the nurse and PA club instructors in individual interviews at their respective schools; and ask the nurse and PA club instructors about satisfaction with intervention, its feasibility, problems meeting objectives, and recommendations for improvement; and record comments.

### Sample size determination and data analysis

#### Power calculations

Sample size calculations incorporated school-based research power approaches [65,66] and were informed by the work done in our pilot study and other studies using MVPA as a primary outcome [67,68]. We assumed a small effect size of 0.20 and an intraclass correlation coefficient (ICC) of 0.02, as noted from the Trial for Activity in Adolescent Girls [67]. Assuming a standard deviation of 80 for minutes of MVPA over 7 days [68], this effect size of 0.20 corresponds to a difference of 16 minutes/week between groups. Given that girls receiving the intervention are encouraged to accumulate at least 60 minutes/day of MVPA, this mean difference is reasonable to expect with a control group that consists of a number of girls who may have never been involved in any organized MVPA due to obstacles, such as limited free or low-cost programs [69]. Using this effect size and ICC, 24 schools (12 intervention) with 50 girls per school would provide power .80 and alpha = 0.05 for two-tailed comparison between two groups. To allow for 20% attrition (12 girls per school) [36,69-71], we plan to recruit 62 girls per school each year.

#### Data analysis

Data will be analyzed using SAS 9.3 (SAS Institute Inc., Cary, NC) [72]. Outcome measures include the primary outcome: minutes of MVPA; secondary outcomes: CV fitness, BMI, and percent body fat; and mediating cognitive and affective variables. Potential covariates measured at baseline are personal factors and behaviors. To adjust for non-independence of observations within each school and the dependence within person, analyses below will be carried out using a multilevel model approach frequently used in group randomized trials [73] that will incorporate baseline measures as covariates [74,75]. Multilevel generalized linear models [73,76] will be fit for ordinal, and discrete outcomes using appropriate link functions. Assumptions of normality for continuous outcome variables will be evaluated; normalizing transformations or generalized linear mixed effect models will be used if warranted [76].

#### Preliminary analyses

Descriptive statistics, such as means and proportions, will be provided for outcomes and baseline measures. Pattern of missing data will be evaluated and missing at random mechanism will be assumed [77]. We will collect information on participants who dropped out and baseline characteristics of those who dropped out will be compared by group to provide information of any potential attrition bias.

#### Primary analyses

Primary analysis is of intention to treat type [78,79], which gives a conservative estimate of treatment effect compared with what would be expected if there was full compliance [80]. Effect sizes and their confidence intervals (adjusted for baseline values of outcomes and nested study design) will be computed. Variance components (school and participants) will be estimated.

We will use: Statistical Model #1: Outcome (after 17 weeks) = outcome (baseline) + study group + covariates. The essential parameter tested in this model is the coefficient for the study group variable. Covariates will be included if preliminary analyses reveal group differences at baseline despite randomization. We will model 9-month post-intervention F/U MVPA, controlling for MVPA after 17 weeks (immediately post-intervention): MVPA (9-month post-intervention F/U) = MVPA (after 17 weeks) + study group + covariates.

#### Secondary analyses

Secondary analyses will be conducted to examine if the primary outcome is mediated by the cognitive and affective variables. Following the approach by MacKinnon and others [81-87], we will conclude that cognitive or affective variables are potential mediators if they change the estimate of intervention effect once included in the model. To test for mediation effect, Statistical Model #1 will be used with MVPA (outcome measure) and with each of the potential mediators. We will refer to Models as Model #1a: MVPA (after 17 weeks) = MVPA (baseline) + study group + covariates; and Model #1b: Mediator variable (after 17 weeks) = Mediator variable (baseline) + study group + covariates. In addition to Models #1a and #1b, the following Statistical Model #2: will be fit: MVPA (after 17 weeks) = MVPA (baseline) + study group + mediator variable (after 17 weeks) + covariates. To explore mediation, we will use the “Causal Steps Method” [88] and evaluate significance of group in Models #1a and #1b, and mediator in Model #2. Effect sizes will be calculated using estimates for regression coefficients and standard errors. Percent of variation mediated will be determined [89].

## Discussion

The objective of this intervention is to increase MVPA as a means to address the high overweight and obesity prevalence among adolescent girls living in low SES urban areas [90]. Process evaluation data from our prior pilot study indicated that the program elements were feasible in a school and acceptable to girls of middle school age, nurses, and after-school PA club instructors. This next step, which involves a group randomized trial, is critical for testing the efficacy of the intervention with a large sample of girls. The multi-year group randomized trial will assist us in refining the intervention components and identifying important theoretical mediators influencing girls’ MVPA. Because many studies with adolescents have included self-report of MVPA [32], this study, which includes objective measures, a large sample size, and an iPad-delivered survey with voiceovers, is expected to shed new light on the relationships among the variables. The findings and process evaluation data will provide important information for researchers to consider when designing interventions to increase girls’ MVPA. If efficacious, our next goal will be to disseminate the intervention in school settings.

## Abbreviations

ACT: Active by Choice Today; BMI: Body mass index; BREQ-2: Behavioral Regulation in Exercise Questionnaire – 2; CV: Cardiovascular disease; F/U: follow-up; HPM: Health Promotion Model; ICC: Intraclass correlation coefficient; MITI: Motivational Interviewing Treatment Integrity; MVPA: moderate to vigorous physical activity; PA: physical activity; PACER: Progressive Aerobic Cardiovascular Endurance Run; SDT: Self-Determination Theory; SES: Socioeconomic status.

## Competing interests

The authors declare that they have no competing interests.

## Authors’ contribution

LBR, KAP, KR, LA, and SMW were responsible for the conception and design of the study and the development of the study protocols. LBR, the principal investigator, wrote the first draft of this manuscript and the final version. ZY contributed to the writing of the analysis section. AV assisted in various ways related to the motivational, individually tailored counseling component of the intervention. KAP, KR, AV, and SMW performed at least one critical revision of the manuscript, and all authors reviewed and approved the final version.

## Pre-publication history

The pre-publication history for this paper can be accessed here:

http://www.biomedcentral.com/1471-2458/13/474/prepub
